# Continuous furosemide does not prevent cardiopulmonary bypass-related acute kidney injury in minimally invasive cardiac surgery: the randomized furosemide trial

**DOI:** 10.1051/ject/2025042

**Published:** 2025-12-17

**Authors:** Tomohisa Takeichi, Yoshihisa Morimoto, Akitoshi Yamada, Takanori Tanaka, Kunihiro Fujiwara, Masanobu Sato, Ryo Toma, Kiyoto Mitsui, Takumi Sugita, Hiroki Yamada, Kunio Gan

**Affiliations:** 1 Department of Clinical Engineering, Kitaharima Medical Center 926-250, Ichiba-cho Ono-shi Hyogo 675-1392 Japan; 2 Cardiovascular Surgery, Kitaharima Medical Center 926-250, Ichiba-cho Ono-shi Hyogo 675-1392 Japan

**Keywords:** Continuous furosemide, Cardiopulmonary bypass (CPB), Minimally invasive cardiac surgery, Randomized controlled trial

## Abstract

*Objectives*: This study aimed to assess whether continuous furosemide administration during cardiopulmonary bypass (CPB) in minimally invasive cardiac surgery (MICS) reduces the incidence of cardiac surgery-associated acute kidney injury (AKI). *Methods*: A total of 100 patients undergoing MICS with CPB were randomly assigned to receive either continuous furosemide infusion or no continuous furosemide during CPB. The primary endpoint was the incidence of AKI. Secondary endpoints included the cardiac surgery-associated neutrophil gelatinase-associated lipocalin (CSA-NGAL) score, urine output within 12 h postoperatively, postoperative furosemide dose requirements, red blood cell transfusion volume, PaO_2_/FiO_2_ ratio, duration of mechanical ventilation, length of stay in the intensive care unit (ICU) and hospital, and in-hospital mortality. *Results*: AKI occurred in 8 patients (16%) in the continuous furosemide group and in 6 patients (12%) in the non-continuous group (relative risk, 0.72; 95% CI, 0.23–2.23). Among the secondary endpoints, urine output within the first 3 h postoperatively and the PaO_2_/FiO_2_ ratio were significantly higher in the continuous furosemide group. However, subgroup analyses revealed no significant differences between the two groups. *Conclusions*: Continuous furosemide administration during CPB did not effectively reduce the incidence of AKI. However, it was associated with a significant increase in postoperative urine output and an improvement in the PaO_2_/FiO_2_ ratio.

## Introduction

Acute kidney injury (AKI) following cardiac surgery involving cardiopulmonary bypass (CPB) is a well-documented complication [1]. Over the past decade, the standardization of AKI definitions has facilitated advancements in diagnosis, treatment, and prevention strategies [2]. Furthermore, the development of urinary biomarkers, such as neutrophil gelatinase-associated lipocalin (NGAL), has enabled early detection of AKI [3].

To mitigate AKI risk, extensive research has been conducted, and the “Kidney Disease: Improving Global Outcomes” (KDIGO) guidelines recommend the implementation of various supportive interventions in high-risk patients [4–6]. 2024 EACTS/EACTA/EBCP guidelines on cardiopulmonary bypass in adult cardiac surgery suggested that the oxygen delivery strategy is part of an important indicator of preventing postoperative AKI [7]. In recent years, the utilization of the RenalGuard (RG) system (RenalGuard Solutions Inc., Milford, MA, USA) has demonstrated efficacy in reducing AKI incidence. This system has been shown to decrease AKI occurrence by 60–75%. The RG system employs forced diuresis through the administration of low-dose furosemide (0.25–0.5 mg/kg), while simultaneously infusing intravenous (IV) fluids in real-time to match urine output, thereby preventing inadvertent volume depletion [8]. From 2011, the system was utilized to prevent contrast-induced acute kidney injury, particularly in high-risk patients such as those with chronic kidney disease (CKD) undergoing contrast media procedures [9–11]. In January 2024, the United States FDA granted a “Breakthrough Device Designation” for RenalGuard therapy in patients undergoing cardiac surgery.

Prior to our retrospective study, intraoperative administration of furosemide during CPB was observed to attenuate AKI development [12]. Effective urinary management during CPB is paramount in AKI prevention [13, 14]. In an experimental rat model of AKI, furosemide administration was associated with reduced apoptotic activity in the renal medulla and cortex compared to non-treated groups [15]. However, continuous furosemide infusion during CPB has not yet been established as a standardized preventive strategy for AKI. Additionally, the direct correlation between increased urine output and AKI prevention remains uncertain. In this study, we investigated the effects of continuous furosemide administration during CPB and demonstrated its potential to mitigate AKI incidence.

## Methods

The clinical trial received approval from the Institutional Review Board of Kitaharima Medical Center on November 4, 2022. Data on preoperative and intraoperative parameters, as well as early postoperative outcomes, were prospectively recorded in the hospital’s electronic medical record system and subsequently analyzed retrospectively. Ethical approval was granted by the local ethics committee (study approval #04-33). Written informed consent was obtained from all participants prior to their inclusion in the study.

### Study design and participants

This study was a prospective, randomized, single-blind trial conducted at a single center, enrolling patients undergoing cardiopulmonary bypass (CPB). Eligible participants were adults aged 20 years or older scheduled for minimally invasive cardiac surgery (MICS). Exclusion criteria included open sternotomy cases, severe chronic kidney disease (estimated glomerular filtration rate [eGFR] < 30 mL/min/1.73m^2^), preoperative hemodialysis dependency, intraoperative hypothermia below 30 °C, preoperative left ventricular ejection fraction (EF) < 30%, emergency procedures, preoperative NGAL levels exceeding 50 ng/mL, unforeseen conversion to open sternotomy, and cases where maintaining a cardiac index (CI) > 2.0 L/min/m^2^ proved challenging, including unexpected bleeding and poor at venous drainage during CPB.

### Randomization and intervention

Patients were randomly assigned to either the continuous furosemide infusion group or the non-continuous furosemide group. Randomization was performed using simple randomization with a random number generator in Microsoft Excel 2019 (Microsoft Corp, Redmond, WA). Only perfusionists involved in the surgical process were informed of the allocated intervention, while other medical personnel, including surgeons, anesthesiologists, and nursing staff, remained blinded. Hemodynamic management during CPB was at the discretion of the perfusionist.

In the continuous furosemide group, a 10 mg bolus of furosemide was administered immediately upon CPB initiation, followed by a continuous infusion at 10 mg/h until CPB termination. The infusion solution comprised 50 mg of furosemide mixed with 45 mL of 20% mannitol. In the non-continuous group, a single 10 mg bolus of furosemide was administered upon CPB initiation, with no further doses permitted in either group. The priming solution used 1000 mL of bicarbonate ringer’s solution. CI was maintained between 2.0 and 2.4 L/min/m^2^, and mean arterial blood pressure (mABP) was controlled between 60 and 80 mmHg. Core body temperature was maintained at either 30.0 °C or 31.0 °C. Hemoglobin (Hb) levels were managed to remain above 8.0 g/dL, with transfusions administered when levels fell below this threshold. To ensure uniform postoperative management, ICU staff remained blinded to CPB intervention details.

CPB was established using a femoral venous cannula (MICS Cannulae; LivaNova, Tokyo, Japan) and a femoral arterial cannula (PCKC-A, MERA, Tokyo, Japan), with circulation maintained by the Heart Assist System III (HAS III, Mera Corporation, Tokyo, Japan). The extracorporeal circuit incorporated a centrifugal pump (MERA Centrifugal Pump HCF-MP23, SENKO MEDICAL INSTRUMENT, Inc., Tokyo, Japan). The extracorporeal circuit is coated with heparin and silicone, while the venous reservoir is treated with SEC1 polymer coating (Terumo Corp., Tokyo, Japan). Blood gas parameters were continuously monitored using a CDI Blood Parameter Monitoring System 500 (Terumo, Tokyo, Japan), which was recalibrated every 60 min, alongside arterial blood gas analyses performed at the same interval. Postoperatively, if urine output fell below 100 mL/h within the first 12 h, a 10 mg bolus of furosemide was administered.

### Outcomes and follow-up

The primary endpoint was the incidence of AKI, as defined by the Kidney Disease: Improving Global Outcomes (KDIGO) criteria. AKI stage 1 was defined as a serum creatinine increase of 1.5–1.9 times baseline or an absolute increase of ≥0.3 mg/dL; stage 2 was defined as a 2.0 to 2.9-fold increase from baseline; and stage 3 was defined as a ≥3.0-fold increase from baseline or the initiation of renal replacement therapy. To focus specifically on CPB-associated AKI, only peak serum creatinine values within the first 48 postoperative hours were analyzed. Baseline creatinine levels were measured preoperatively, and AKI classification was based exclusively on serum creatinine changes.

Secondary endpoints included postoperative NGAL levels at 3 h, classified according to the cardiac surgery-associated NGAL (CSA-NGAL) score: tubular damage unlikely (<50 ng/mL), possible tubular damage (50–150 ng/mL), tubular damage (150–1000 ng/mL), and severe tubular damage (>1000 ng/mL). Additional secondary outcomes included 12-hour postoperative urine output, postoperative furosemide dosing, nadir mABP, incidence of low cardiac output syndrome (LCOS), number of red blood cell (RBC) transfusions, PaO_2_/FiO_2_ ratio, duration of mechanical ventilation (hours), ICU and hospital length of stay, and in-hospital mortality.

### Statistical analysis

Based on prior data, a 50% incidence of AKI was anticipated in the control group. A previous study on continuous furosemide administration in cardiac surgery patients suggested a potential absolute AKI risk reduction of 50%. To achieve 80% power with a two-sided *α* level of 0.05, a sample size of 48 patients per group was required. Allowing for an estimated 20% dropout rate, the target enrollment was set at 116 participants, with a planned total study population of 200 patients.

Continuous variables were presented as mean ± standard deviation (SD). Between-group comparisons were conducted using either an unpaired Student’s *t*-test for parametric data or a Mann-Whitney *U-*test for nonparametric data, as appropriate. Categorical variables were expressed as frequencies (%) and analyzed using Pearson’s chi-square test. Relative risks or odds ratios, along with 95% confidence intervals (CIs), were calculated for key endpoints, including AKI incidence and CSA-NGAL classifications. Pearson correlation coefficients were employed to assess relationships between creatinine increase rates and urine output. Prespecified subgroup analyses were conducted to evaluate potential effect modifications of continuous furosemide therapy, utilizing logistic regression models and two-way analysis of variance. All statistical analyses were performed using Microsoft Excel 365.

## Results

**Figure 1:**
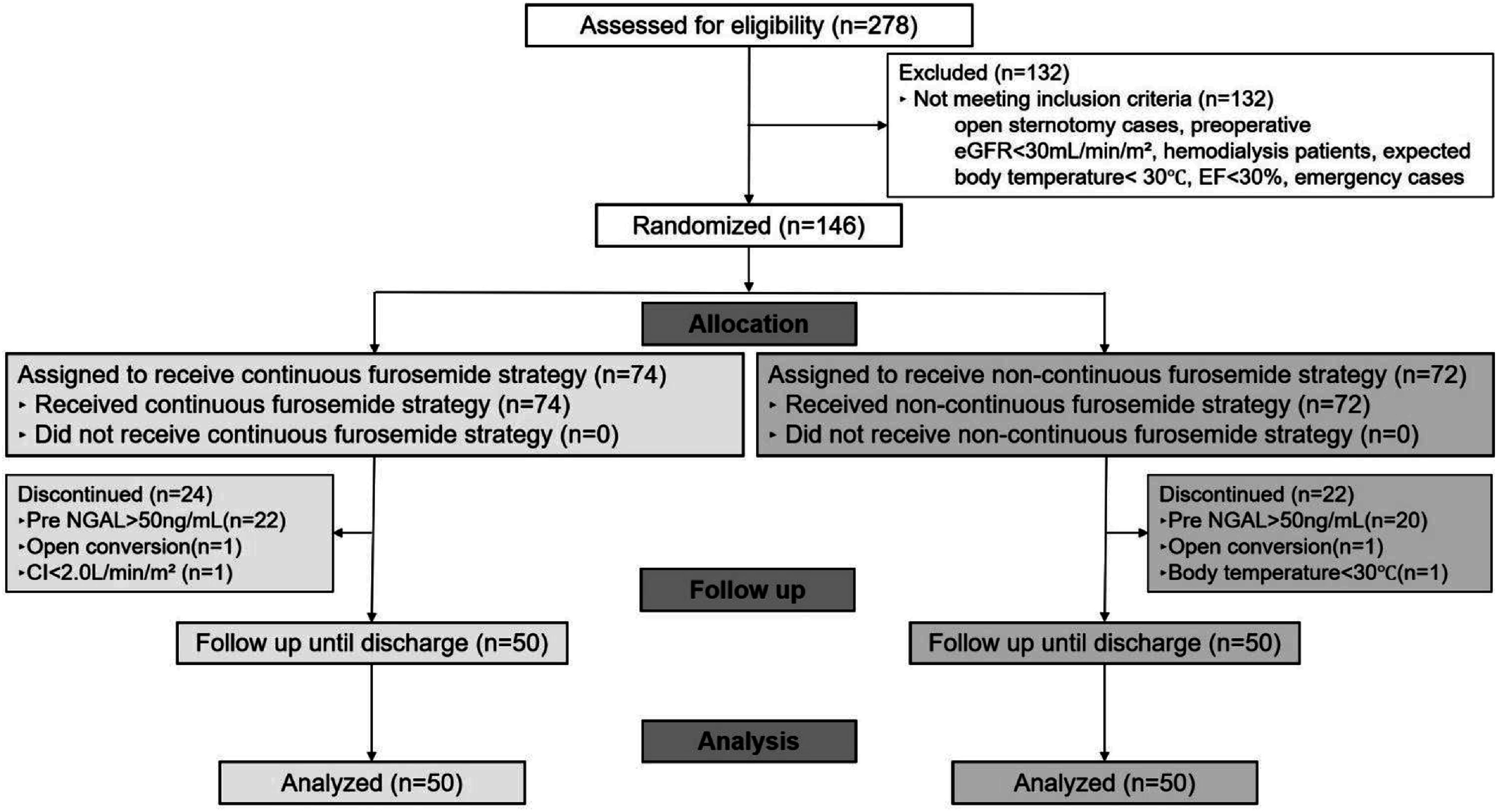
Flow diagram of patient selection for a single-center RCT. eGFR = Estimated glomerular filtration rate; EF = ejection fraction; NGAL = neutrophil gelatinase-associated lipocalin; CI = cardiac index.

A total of 278 patients were assessed for eligibility between November 2022 and December 2024, of whom 146 were randomized (Figure 1). Among them, 46 patients received the allocated CPB management but discontinued the intervention due to a preoperative NGAL value exceeding 50 ng/mL, unforeseen conversion to open sternotomy, unanticipated complications, or challenges in maintaining a CI below 2.0 L/min/m^2^. Consequently, the final study cohort comprised 100 participants, with 50 in the continuous furosemide strategy group and 50 in the non-continuous furosemide group.

Baseline characteristics were comparable between the two groups (Table 1). Operative details are summarized in Table 2. Patients in the continuous furosemide group exhibited significantly higher urine output during CPB (*P* = 0.001), after CPB (*P* = 0.001), and overall (*P* = 0.006), as well as a greater total furosemide dose (*P* = 0.001). Although there was no statistically significant difference in overall fluid balance, a trend toward reduction was observed.

**Table 1:** Baseline characteristics of patients. Definition of abbreviations: eGFR = Estimated glomerular filtration rate; EF = ejection fraction; NGAL = neutrophil gelatinase-associated lipocalin. Data represent mean ± SD, number (%).

	Continuous (n = 50)	Non-Continuous (n = 50)	*P* value
Age (years)	65.8 ± 13.5	69.4 ± 11.6	0.213
Female sex, *n* (%)	18 (36.0)	20 (40.0)	0.681
Weight (kg)	57.1 ± 11.2	58.3 ± 13.8	0.424
Body mass index (kg/m^2^)	20.8 ± 4.1	22.1 ± 3.6	0.107
Hypertension, *n* (%)	19 (38.0)	20 (40.0)	0.838
Diabetes, *n* (%)	17 (7.3)	21 (8.8)	0.561
Creatinine (mg/dL)	0.89 ± 0.25	0.94 ± 0.32	0.510
eGFR (mL/min/1.73m^2^)	62.4 ± 19.6	62.3 ± 15.6	0.950
Stage of CKD			
G1 (eGFR > 90 mL/min/1.73 m^2^)	4 (8.0)	4 (8.0)	1.000
G2 (eGFR 60–89 mL/min/1.73 m^2^)	22 (44.0)	27 (54.0)	0.317
G3a (eGFR 45–59 mL/min/1.73 m^2^)	14 (28.0)	13 (26.0)	0.822
G3b (eGFR 30–44 mL/min/1.73 m^2^)	10 (20.0)	6 (12.0)	0.275
Urine NGAL (ng/mL)	15.5 ± 9.5	14.8 ± 8.8	0.967
LVEF (%)	60.9 ± 10.0	59.6 ± 8.8	0.288
EuroSCOREII	2.3 ± 2.1	2.6 ± 2.2	0.532

**Table 2:** Operative details of patients. Definition of abbreviations: CPB = cardiopulmonary bypass; PI = perfusion index; SvO_2_ = venous oxygen saturation; mABP = mean arterial blood pressure; NGAL = neutrophil gelatinase-associated lipocalin. Data represent mean ± SD, number (%).

	Continuous (*n* = 50)	Non-continuous (*n* = 50)	*P* value
Surgical procedure			
Single valve, *n* (%)	38 (76.0)	40 (80.0)	0.639
Double valve, *n* (%)	6 (12.0)	6 (12.0)	1.000
Aortic replacement, *n* (%)	1 (2.0)	0	0.496
Adult congenial, *n* (%)	3 (6.0)	2 (4.0)	1.000
Cardiac tumor, *n* (%)	2 (4.0)	2 (4.0)	1.000
Redo operation, *n* (%)	8 (16.0)	3 (6.0)	0.111
CPB time (min)	205.2 ± 50.3	226.9 ± 70.2	0.147
Cross clamp time (min)	124.1 ± 40.2	142.9 ± 50.6	0.095
Nadir rectal temperature (°C)	30.5 ± 1.1	30.7 ± 0.76	0.562
Median PI (L/min/m^2^)	2.1 ± 0.13	2.1 ± 0.14	0.293
Median SvO_2_ (%)	85.7 ± 4.7	85.4 ± 3.2	0.971
Median mABP (mmHg)	67.3 ± 7.9	65.3 ± 9.2	0.762
Fluid balance			
During CPB (mL/kg)	24.6 ± 34.8	33.3 ± 40.5	0.324
Overall (mL/kg)	51.2 ± 37.3	69.6 ± 56.2	0.067
Urine output			
Before CPB (mL/kg)	0.91 ± 1.5	1.2 ± 2.0	0.678
During CPB (mL/kg/h)	14.2 ± 6.7	10.0 ± 5.9	0.001
After CPB (mL/kg)	12.5 ± 8.4	6.7 ± 7.1	0.001
Overall (mL/kg)	60.0 ± 29.1	46.4 ± 28.3	0.006
Total Furosemide dose (mg)	44.1 ± 8.3	4.2 ± 4.9	0.001
Urine NGAL during CPB (ng/mL)	12.4 ± 6.7	13.6 ± 10.0	0.354

### Endpoints

**Figure 2:**
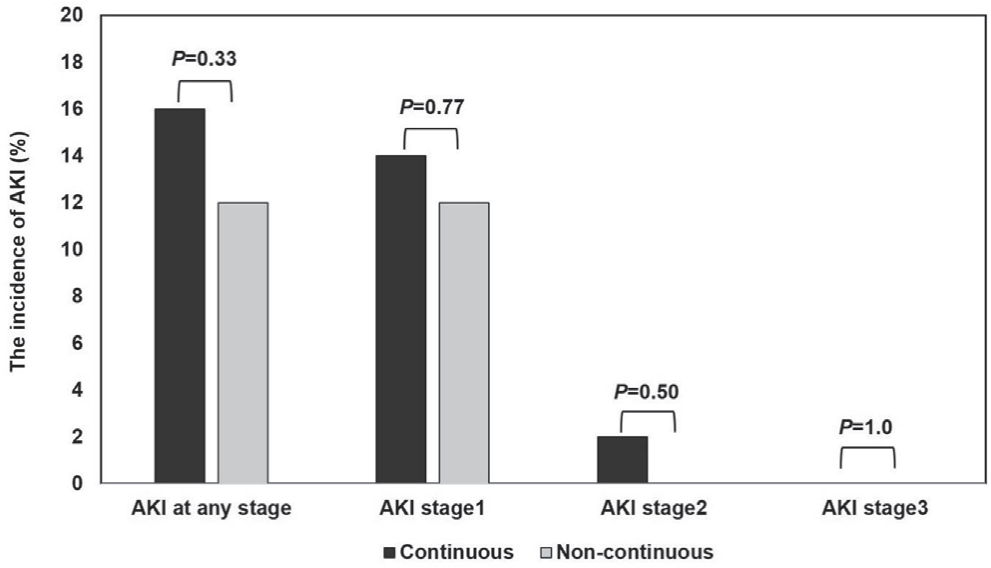
The incidence of AKI among continuous furosemide and non-continuous furosemide methods. AKI = acute kidney injury.

The number of patients included in the primary and secondary endpoint analyses is presented in Table 3. AKI occurred in 8 patients (16%) in the continuous furosemide group and in 6 patients (12%) in the non-continuous group (relative risk, 0.72; 95% CI, 0.23–2.23). In terms of AKI staging, stage 1 AKI was observed in 7 of 50 patients (14.0%) in the continuous furosemide group and in 6 of 50 patients (12%) in the non-continuous group (odds ratio, 0.72; 95% CI, 0.229–2.23). Stage 2 AKI occurred in 1 patient (2.0%) in the continuous furosemide group and was not observed in the non-continuous group (odds ratio, 0.84; 95% CI, 0.26–2.69). No cases of stage 3 AKI were reported (odds ratio, 0.33; 95% CI, 0.013–8.21) (Figure 2, Table 3).

**Table 3:** Primary and secondary endpoints. Definition of abbreviations: AKI = acute kidney injury; NGAL = neutrophil gelatinase-associated lipocalin; Cr = creatinine; LCOS = Low cardiac output syndrome; mABP = mean arterial blood pressure; RBC = red blood cell; ICU = intensive care unit. Data represent mean ± SD, number (%), RR[CI].

	Continuous (*n* = 50)	Non-continuous (*n* = 50)	Relative risk [95 %CI] or odd ratio [95%CI]	*P* value
Development of AKI	8 (16.0)	6 (12.0)	0.72 [0.23 to 2.23]	0.330
AKI stage 1, *n* (%)	7 (14.0)	6 (12.0)	0.84 [0.26 to 2.69]	0.766
AKI stage 2, *n* (%)	1 (2.0)	0	0.33 [0.013 to 8.21]	0.496
AKI stage 3, *n* (%)	0	0	1.0 [0.019 to 51.38]	1.000
Urine NGAL after cardiac surgery (ng/mL)	71.7 ± 103.8	118.0 ± 291.1		0.380
CSA-NGAL score				
Tubular damage unlikely, *n* (%)	31 (62.0)	29 (58.0)	0.85 [0.37 to 1.88]	0.683
Tubular damage possible, *n* (%)	12 (24.0)	14 (28.0)	1.2 [0.50 to 3.01]	0.648
Tubular damage, *n* (%)	7 (14.0)	5 (10.0)	0.68 [0.20 to 2.31]	0.538
Severe tubular damage, *n* (%)	0	2 (4.0)	5.2 [0.24 to 111.2]	0.495
NGAL increasing rate (%)	426.5 ± 795.7	992.5 ± 2905.8		0.437
Maximum serum Cr increasing rate within 48 h after surgery (%)	11.0 ± 29.0	9.0 ± 20.9		0.891
Urine output after surgery				
0–3 h (mL/kg/h)	10.0 ± 4.4	7.4 ± 4.1		0.002
3–6 h (mL/kg/h)	3.3 ± 2.2	3.0 ± 1.5		0.756
6–9 h (mL/kg/h)	1.6 ± 1.1	1.5 ± 0.84		0.684
9–12 h (mL/kg/h)	1.1 ± 0.92	1.2 ± 1.1		0.475
Furosemide dose rate after cardiac surgery, *n* (%)	4 (8.0%)	6 (12.0%)		0.505
Nadir mABP (mmHg)	54.3 ± 8.3	53.2 ± 7.5		0.761
Postoperative LCOS, *n* (%)	0	0		1.000
No. of RBC units (u)	2.4 ± 2.8	2.3 ± 3.1		0.582
PaO_2_/FiO_2_ ratio	325.8 ± 104.5	277.1 ± 110.9		0.009
Intubation time (h)	7.0 ± 5.4	9.5 ± 12.4		0.271
Length of ICU stay (d)	1.0 ± 0.49	1.4 ± 1.6		0.078
Length of postoperative hospital stay (d)	10.2 ± 6.1	9.6 ± 3.9		0.717
Hospital mortality, *n* (%)	0	0		1.000

**Figure 3:**
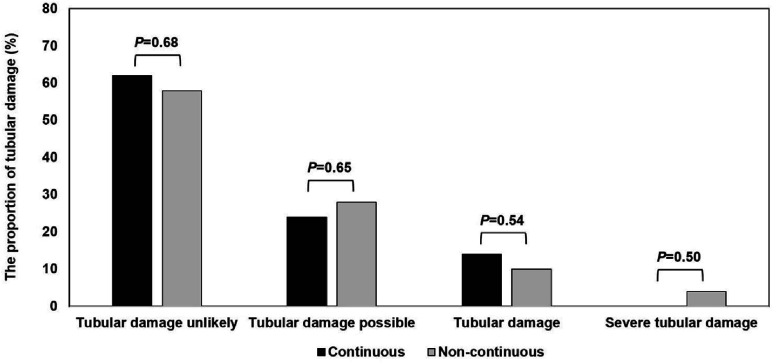
The proportion of tubular damage compared to the continuous furosemide and non-continuous furosemide. Postoperative NGAL levels at 3 h, classified according to the cardiac surgery-associated NGAL (CSA-NGAL) score. Tubular damage unlikely is less than 50 ng/mL. Tubular damage possible is 50–50 ng/mL. Tubular damage is 150–1000 ng/mL. Severe tubular damage is more than 1000 ng/mL.

Among the secondary endpoints, urine output within 12 h postoperatively (0–3 h) (*P* = 0.002) and the PaO_2_/FiO_2_ ratio (*P* = 0.009) were significantly higher in the continuous furosemide group. Although the length of ICU stay did not differ significantly between groups, there was a trend toward a shorter duration in the continuous furosemide group (*P* = 0.078). Other parameters, including the NGAL value at 3 h postoperatively, CSA-NGAL score, postoperative furosemide dose rate, number of RBC transfusions, duration of intubation, and total length of ICU and hospital stay, as well as in-hospital mortality, showed no significant differences between the groups (Figure 3, Table 3).

### Correlation between urine output and creatinine increase

In the continuous furosemide group, a significant negative correlation was found between creatinine increase and urine output during CPB at 60–120 min (*r* = −0.299, *P* = 0.038) and post-CPB urine output (*r* = −0.385, *P* = 0.006). Weak negative correlations were observed between creatinine increase and both total urine output during CPB and urine output at 6–9 h postoperatively. Urine output during CPB (0–60 min, 120–180 min), total urine output after surgery, and postoperative urine output at 3–6 h and 9–12 h were not significantly correlated with creatinine increase.

Conversely, in the non-continuous furosemide group, a significant negative correlation was noted between creatinine increase and urine output during CPB at 60–120 min (*r* = −0.421, *P* = 0.024). Weak negative correlations were also observed for urine output during CPB at 120–180 min, total urine output during CPB, total postoperative urine output, and urine output at 9–12 h postoperatively. No significant correlation was found between creatinine increase and urine output during CPB at 0–60 min, post-CPB urine output, or urine output at 0–3 h, 3–6 h, and 6–9 h postoperatively (Table 4).

**Table 4:** Correlation between urine output and Cr increasing rate. Definition of abbreviations: CPB = cardiopulmonary bypass.

	Continuous (*n* = 50)		Non-continuous (*n* = 50)	
	*r*	*P* value	*r*	*P* value
Urine output during CPB				
0–60 min (mL/kg)	−0.226	0.118	−0.153	0.288
60–120 min (mL/kg)	−0.299	0.038	−0.421	0.024
120–180 min (mL/kg)	−0.243	0.173	−0.311	0.065
Urine output after CPB (mL/kg)	−0.385	0.006	−0.167	0.246
Total urine output during CPB (mL/kg/h)	−0.244	0.090	−0.384	0.050
Overall urine output (mL/kg)	−0.144	0.318	−0.268	0.062
Urine output after cardiac surgery				
0–3 h (mL/kg/h)	−0.393	0.005	−0.210	0.143
3–6 h (mL/kg/h)	−0.122	0.398	−0.205	0.153
6–9 h (mL/kg/h)	−0.274	0.054	−0.111	0.442
9–12 h (mL/kg/h)	0.097	0.501	−0.273	0.057

### Subgroup analysis

There were no significant differences between the continuous and non-continuous furosemide groups regarding age, sex distribution, presence of renal impairment, EuroSCORE, rate of redo operations, and CPB time (Table 5).

**Table 5:**
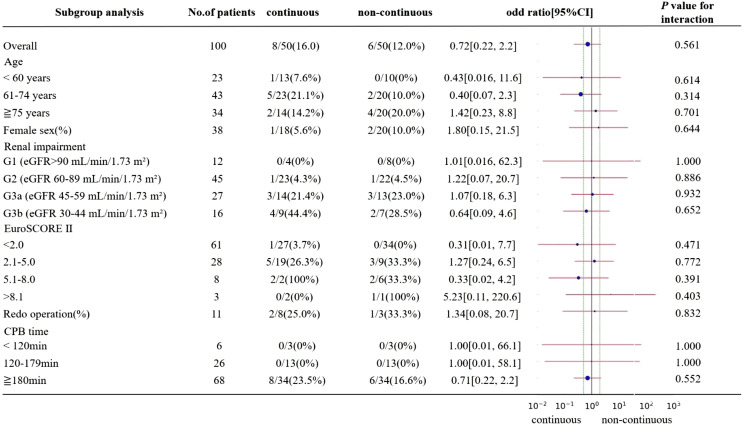
Subgroup analyses of the primary endpoint. The vertical lines indicate the overall estimate (gray solid line) and the 95% CI (green dashed lines) for the primary endpoint as calculated for the entire analysis cohort.

## Discussion

Response to furosemide has been effectively utilized to predict the progression of AKI based on urine volume responsiveness [16, 17]. Additionally, while several studies have explored its potential preventive effects on AKI, a definitive consensus has not been reached [8, 9, 15]. The importance of appropriate intravascular volume management in AKI prevention has already been emphasized by the KDIGO group as one of the most crucial strategies [6].

In this RCT, we investigated whether a continuous furosemide strategy during CPB, as opposed to an intermittent furosemide approach, could reduce postoperative AKI in patients undergoing cardiovascular surgery. While continuous furosemide administration did not significantly lower AKI incidence, it led to a substantial increase in urine output during CPB, post-CPB, and overall. Furthermore, postoperative urine output within the first 3 h was significantly higher, accompanied by an improved PaO_2_/FiO_2_ ratio. Although ICU length of stay did not differ significantly between groups, there was a trend toward a shorter stay in the continuous furosemide group. Other parameters, including NGAL levels at 3 h postoperatively, CSA-NGAL scores, postoperative furosemide dosage, red blood cell transfusions, intubation time, hospital length of stay, and in-hospital mortality, did not differ significantly between groups.

Analysis of urine output and Cr increase rates revealed a significant negative correlation in the continuous furosemide group between urine output at 60–120 min during and after CPB and the Cr increase rate. A weaker negative correlation was observed between the Cr increase rate and total urine output during CPB, as well as urine output 6–9 h postoperatively. In the intermittent furosemide group, a significant negative correlation was observed between urine output at 60–120 min during CPB and the Cr increase rate, while a weaker correlation was found with urine output at 120–180 min during CPB, total urine output, and urine output at 9–12 h postoperatively. Kunt et al. showed that after ICU admission, a comparison between bolus and continuous infusion of furosemide revealed that postoperative serum creatinine levels increased in both groups. However, the maximum postoperative creatinine clearance significantly decreased in the bolus group, suggesting a greater decline in renal function compared to the continuous infusion group. Continuous infusion appeared to support a beneficial sequence – stable urine output, prevention of volume overload, maintenance of creatinine clearance, and a reduced need for renal replacement therapy – ultimately indicating its effectiveness in preventing AKI [18]. Also, Luckraz et al. reported that the RG system, which facilitates forced diuresis using furosemide at a rate of 10 mg/h, significantly reduced AKI incidence compared to the control group. The RG system was implemented from the induction of anesthesia to 6 h postoperatively. It is hypothesized that early and sustained diuresis promotes the elimination of toxic substances – such as harmful metabolic byproducts, inflammatory cytokines, and oxidative stress-related compounds – by preventing their accumulation in the kidneys and facilitating their early excretion. The maintenance of continuous urine output may also prevent intratubular concentration and cast formation, thereby suppressing the progression of renal injury and contributing to the prevention of AKI (8). An in vitro study demonstrated that furosemide administration resulted in reduced apoptosis in the renal medulla and cortex compared to non-administration. Furosemide is secreted by the proximal tubule via the organic anion transport system and exerts its effect by inhibiting the Na^+^-K^+^-2Cl^−^ cotransporter in the luminal side of the ascending loop of Henle, thereby reducing NaCl reabsorption. Despite the low partial oxygen pressure (10–15 mmHg) in the renal medulla, its energy demand remains high. It is hypothesized that furosemide mitigates metabolic activity in the loop of Henle through its pharmacological effects [15]. However, a systematic review and meta-analysis concluded that while the RenalGuard system appears to be a promising tool for the prevention of AKI, the current evidence is insufficient to make definitive recommendations. The authors emphasize the need for larger, well-designed randomized controlled trials, particularly to assess the risk-benefit profile of its use in high-risk patient populations [19]. Also, a systematic review reported that furosemide does not reduce AKI incidence. On the other hand, ventilation time was significantly shorter [20]. In this RCT, the preventive effect of continuous furosemide during CPB against AKI was not confirmed. AKI is a multifactorial condition involving hemodynamic instability, renal ischemia-reperfusion injury, inflammation, and oxidative stress. Simply increasing urine output through diuresis does not address the underlying causes, such as reduced renal perfusion or inflammation [20]. In critical care settings, some studies have suggested that renal responsiveness to intravenous furosemide may serve as an indicator of AKI progression, with low urine output following administration being independently associated with AKI [18, 21]. Notably, a novel finding in our study was the improvement in the postoperative PaO_2_/FiO_2_ ratio in the continuous furosemide group. Additionally, ICU length of stay showed a trend toward reduction. This may be attributed to an overall tendency toward a negative fluid balance and an increase in postoperative urine output within the first 3 h.

Re-expansion pulmonary edema (RPE) is a rare but serious complication following MICS and can significantly impair pulmonary function [22, 23]. The mortality rate associated with acute RPE is approximately 20% [24]. RPE arises due to ischemia-reperfusion injury and inflammatory responses in the lungs [22–24]. Continuous furosemide administration during CPB may potentially attenuate pulmonary function deterioration in cases where RPE occurs. However, in this study, lung compliance post-cardiac surgery was not assessed, limiting further conclusions.

Urine output management has been identified as an important factor in AKI prevention [13]. However, an optimal urine output management strategy during CPB has yet to be established. In this study, urine output was measured every 60 min, including post-CPB values up to 12 h postoperatively, and its correlation with Cr increase rates was evaluated in both groups. A significant negative correlation between urine output at 60–120 min and Cr increase rate was observed in both groups, whereas no correlation was found beyond 120 min. This may be attributable to variations in the timing of aortic de-clamping, as urine output tends to decrease following de-clamping due to cardiac consumption. Khademi et al. similarly reported no significant correlation between intraoperative urine output and postoperative changes in blood urea nitrogen (BUN) or creatinine, consistent with our findings [25]. Therefore, evaluating urine output at 60-minute intervals during CPB is essential.

Regarding post-CPB urine output, in the continuous furosemide group, diuretic effects persisted for up to 3 h postoperatively, demonstrating a negative correlation with Cr increase rates. This suggests that increased urine output immediately after surgery may contribute to AKI prevention, warranting further attention. The RG system has been shown to effectively manage fluid balance, facilitating high urine output while maintaining intravascular volume and minimizing the risks of both overhydration and underhydration [8, 26]. In this study, low cardiac output syndrome (LCOS) was not observed in either group, and no significant differences were noted in nadir mABP postoperatively. However, a graded relationship between the duration of mABP < 55 mmHg and AKI risk has been reported [27]. Optimizing perioperative hemodynamics while ensuring adequate urine output may mitigate AKI risk, and continuous furosemide administration until ICU management may be beneficial.

Finally, continuous furosemide during CPB did not have a preventive effect on AKI. On the other hand, it led to improving postoperative PaO_2_/FiO_2_ by increasing intraoperative and postoperative urine output.

### Study limitations

The primary limitation of this study is its single-center design. Second, AKI was defined solely based on creatinine changes within seven days postoperatively; however, creatinine production is influenced by muscle mass and may vary considerably based on factors such as age, sex, and nutritional status. Additionally, CPB management did not maintain a constant DO_2_, which may have impacted AKI outcomes. Third, urine output during aortic cross-clamping and post de-clamping should be analyzed separately. Finally, this study was not blinded, and the control group may have been subject to the Hawthorne effect.

## Data Availability

All available data are incorporated into the article.
